# Automatic Approach for Lung Segmentation with Juxta-Pleural Nodules from Thoracic CT Based on Contour Tracing and Correction

**DOI:** 10.1155/2016/2962047

**Published:** 2016-11-16

**Authors:** Jinke Wang, Haoyan Guo

**Affiliations:** ^1^Department of Software Engineering, Harbin University of Science and Technology, Rongcheng, China; ^2^School of Computer Science and Technology, Harbin Institute of Technology, Weihai, China

## Abstract

This paper presents a fully automatic framework for lung segmentation, in which juxta-pleural nodule problem is brought into strong focus. The proposed scheme consists of three phases: skin boundary detection, rough segmentation of lung contour, and pulmonary parenchyma refinement. Firstly, chest skin boundary is extracted through image aligning, morphology operation, and connective region analysis. Secondly, diagonal-based border tracing is implemented for lung contour segmentation, with maximum cost path algorithm used for separating the left and right lungs. Finally, by arc-based border smoothing and concave-based border correction, the refined pulmonary parenchyma is obtained. The proposed scheme is evaluated on 45 volumes of chest scans, with volume difference (VD) 11.15 ± 69.63 cm^3^, volume overlap error (VOE) 3.5057 ± 1.3719%, average surface distance (ASD) 0.7917 ± 0.2741 mm, root mean square distance (RMSD) 1.6957 ± 0.6568 mm, maximum symmetric absolute surface distance (MSD) 21.3430 ± 8.1743 mm, and average time-cost 2 seconds per image. The preliminary results on accuracy and complexity prove that our scheme is a promising tool for lung segmentation with juxta-pleural nodules.

## 1. Introduction

Multidetector CT makes chest imaging with high-resolution and submillimeter isotropic characteristics, which greatly promote the automatic analytical techniques on medical imaging. Precise segmentation of pulmonary parenchyma is regarded as a critical step for automatic detection of various lung diseases. However, accurate lung segmentation often failed when abnormity turns up, and abnormity may be missed or other tissues that not belong to lungs could be included. Thus, conventional segmentation techniques are often insufficient to segment pulmonary parenchyma from chest CT datasets.

Previous work on lung segmentation can be roughly classified into two categories. The first category is threshold-based methods, which depend on the different attenuations between lung parenchyma and its surrounding tissues [1–9]. The main limitation of these methods is that their accuracy is badly influenced by pleura abnormity or artifact and often result in oversegmentation. Most of the threshold-based methods are two-dimensional approaches that process each axial section separately. Although it is a reasonable choice for thick slices CT, three-dimensional approach is more preferable when isotropic data is available, in which inconsistency between slices can be avoided.

Sun et al. [1] proposed a fully three-dimensional based lung segmentation and visualization technology. Firstly, in the preprocessing phase, isotropic filtering is used to improve the signal-to-noise ratio; and then, wavelet transform-based interpolation is applied to reconstruct the 3D voxels. Finally, by use of region growing, homogeneity, and gradient features, the lung region is extracted. Brown et al. [2, 3] also suggested a system framework based on 3D region growing and morphology smoothing; moreover, they proposed a semantic network anatomical model. On the basis of the attenuation threshold, shape, adjacent properties, volume, and relative position, the model can simulate the chest wall, mediastinum, bronchial tree, and left and right lungs to distinguish the different anatomical structures. Sun et al. [4] developed a threshold-based segmentation method for missed diagnosis of large tumor. First, a normal shape model of lung is constructed by training of 41 sets of segmented datasets; second, for initialization, rib-based matching algorithm is used to produce the contour. Since the shape model cannot capture the details of the border, thus graph-cut method is implemented for the recovery of the details.

The second category is specific abnormity-based methods, which focus on specific abnormal diseases [10–15]. Due to their specificity on particular case, they are not applicable for routine test of large-scale datasets.

Sofka et al. [10] from Siemens used the visible structure knowledge of chest CT to present a multistage learning method. Firstly, the method identifies the spine among the tracheas; secondly, a hierarchical network is used to predict the posture parameter of left and right lungs. Thirdly, by use of the marks near the ribs and spine, a shape model is initialized and followed by a transformation operation to achieve the refinement. Korfiatis et al. [11] proposed a texture classification-based method for interstitial lung disease. The method used intensity-based *K*-means clustering for initialization, and for containing pixels that around the initial contour, the statistical features of intensity and wavelet coefficients are calculated for support vector classification. In order to compensate for the lost juxta-pleural nodules and ensure the smoothness of the lung boundary, several methods have been proposed to correct the lung contours [12, 13]. Yim and Hong [12] proposed a new curvature-based method for correcting the segmented lung boundary, a 3D branch-based region growing algorithm was utilized to segment the trachea and the left and right bronchi with adaptive growing conditions. Pu et al. [13] developed a lung segmentation method for reducing errors result from juxta-pleural tumor in traditional thresholding approaches. The proposed method begins with segmenting the lung contour with thresholding and smoothing and then flooding in the nonlung region of each slice; by this way, the initial border of the lung is tracked, and the adaptive border marching algorithm is utilized for reincluding the juxta-pleural tumor.

In addition to the above-mentioned studies, a few algorithms focus on diverse lung scans with dense pathologies being proposed. Sluimer et al. [16] proposed an atlas-based technology for lung segmentation with severe lesion. By registering 15 sets of chest CT to referenced lung atlas, the probability atlas is constructed, and then elastic registering is used for mapping the probability atlas to new scans for initialization and transformation. Finally, the trained lung border is utilized for refining the lung border.

The existing methods are either not taking the juxta-pleural tumors into consideration or too specified to be qualified for large-scale testing. Alleviating these difficulties is exactly what we are concerned with in this paper. We developed a fully automatic framework to segment pulmonary parenchyma with juxta-pleural nodules from chest CT. It starts from skin boundary detection with maximum connected component analysis, and then, rough segmentation of lung contour is implemented by diagonal-based tracing, which is followed by the separation of the left and right lungs with maximum cost path algorithm. And the final segmentation of pulmonary parenchyma is achieved by arc-based smoothing and concave-based correction. Our scheme is evaluated on 45 sets of CT scans, and its results are compared with the state of the art method, which is validated by the manual segmentation standard of radiologist.

## 2. Methods

**Figure 1:**
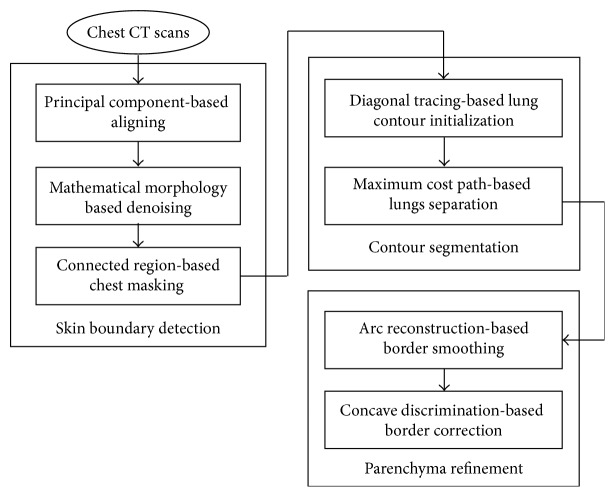
Flowchart of the proposed scheme on lung segmentation.

In this section, the proposed framework will be described in detail. It is a multistep approach that gradually accumulates information until the final result is obtained. We depict the flowchart of the framework in Figure 1. It is subdivided into three phases: skin boundary detection, contour segmentation, and parenchyma refinement. In the rest of the parts, we further describe each individual step and explain how to segment pulmonary parenchyma automatically from chest CT.

### 2.1. Skin Boundary Detection

Skin boundary detection is the foundation of lung segmentation. In view of the high contrast between chest and the background, threshold-based method is utilized for segmentation purpose. In this section, firstly, principal component-based image aligning is implemented to correct the tilted scans; secondly, mathematical morphology operation is applied for noise reduction, and finally, by maximum connected region analyzing, the chest mask is extracted.

#### 2.1.1. Principal Component-Based Image Aligning

**Figure 2:**
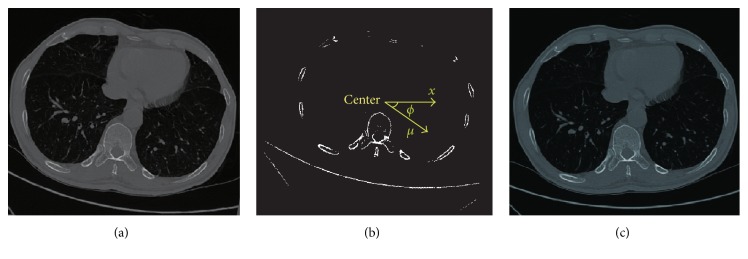
Illustration of image aligning. (a) Original tilted image. (b) Principal component analysis. (c) Aligned image after rotated.

The contour detection algorithm assumes that all patients have the same pose. In particular, it assumes that they lie upright and on their back in the scanner. This assumption is in most cases true due to the standardized CT scanning protocol. However, there are some rare cases in which the patients lie on their side, as shown in Figure 2(a). Because the border detection algorithm is not able to directly handle such scans in view of missing the starting point, an algorithm has been developed which automatically identifies scans in which patients lie on their side and rotates them accordingly.

In this paper, we limit the inclination angle on the *x*-*y* plane, and using the rotation method proposed by [17] for aligning. Firstly, for all the bone voxels on the *x*-*y* plane, principal component analysis [18] is applied for extracting the first principal component *μ*, and then *μ* is mapped to the positive direction of the *x*-axis to generate the rotation matrix *R* with the rotated degree *ϕ*:

(1) $$\begin{array}{c} \phi =\arctan\left(\;\frac{\mu_{2}}{\mu_{1}}\;\right), \end{array}$$

where *μ*_1_ is the mapping of vector *μ* on *y*-axis, while *μ*_2_ is the mapping of vector *μ* on *x*-axis. It is assumed that *μ* is orthogonal to the patients sagittal plane and tangential to his coronal plane. It is further assumed that the angle *ϕ* between *μ* and the positive *x*-axis is between −90° and 90°. If this is not the case, that is, *μ*_1_ < 0, the direction of *μ* is inverted by multiplying −1.

A diagonal-based border detection algorithm is utilized in the subsequent section. By experience only if *ϕ* is out of [−15,15] can the aligning algorithm be applied, or the initial point of lung border could be missed. As *ϕ* is in [−15,15], the influence on the boundary tracking algorithm can be eliminated. As shown in Figure 2, by rotating around the center for *ϕ* degree, the tilted image is aligned.

#### 2.1.2. Mathematical Morphology-Based Denoising

**Figure 3:**
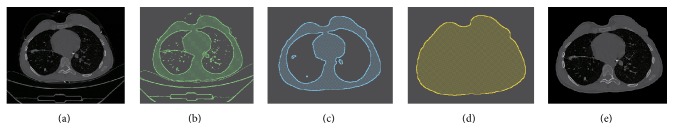
Illustration of skin boundary detection. (a) Original chest CT. (b) Otsu thresholding. (c) Morphological open. (d) Chest mask. (e) Final chest segmentation.

The main problem in skin boundary detection is the existence of various external noises, including human appendant, bed sheet, and CT scanner itself (Figure 3(a)). To eliminate these noises, firstly, Otsu threshold [19] is used for binary processing (Figure 3(b)); secondly, by morphological opening operation, salt noise in the CT scan, bed sheet, and the scanner itself are removed (Figure 3(c)). Finally, by connected regional analysis, the chest mask is determined (Figure 3(d)), and by masking the original chest scan, the final chest region is obtained (Figure 3(e)).

### 2.2. Rough Segmentation of Lung Contour

After skin boundary detection, we step into lung parenchyma segmentation. In this section, two procedures are applied: (1) diagonal tracing-based lung contour initialization; (2) maximum cost path-based lungs separation.

#### 2.2.1. Diagonal Tracing-Based Lung Contour Initialization

A diagonal tracing-based method is proposed for lung contour initialization, with the detailed description in the following.

**Step 1 .**

**Figure 4:**
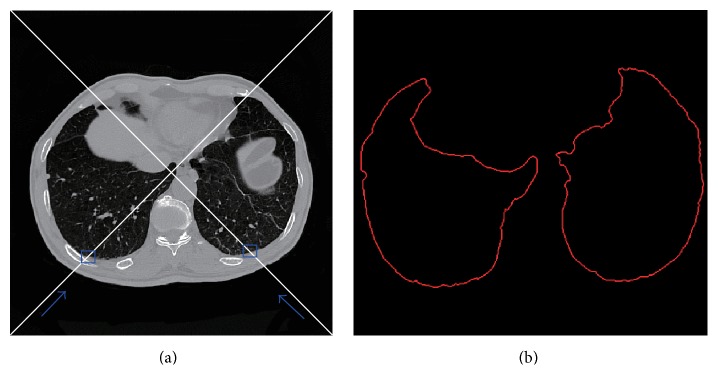
Illustration of diagonal-based contour tracing. (a) Searching the start point along major and minor diagonal. (b) Rough contour after diagonal-based tracing.

Define the major diagonal as the searching path (Figure 4).

**Step 2 .**

Search first *P*_0_ with three consecutive “0s” as the start point of the left lung.

**Step 3 .**

8-neighborhood-based boundary tracing is utilized for boundary extraction of the left lung. Assume the boundary point set is denoted by {*P*_1_(*a*_1_, *b*_1_), *P*_2_(*a*_2_, *b*_2_),…, *P*_*n*−1_(*a*_*n*−1_, *b*_*n*−1_), *P*_*n*_(*a*_*n*_, *b*_*n*_)}.

**Step 4 .**

Once an overlap between the final two points and the initial two points is found, for example, *P*_*n*_ = *P*_2_, *P*_*n*−1_ = *P*_1_, the algorithm ends.

By this way, the boundary of the left lung is achieved; similar to the method of obtaining the left lung border, by searching the start point along the minor diagonal, with the accompanied boundary tracing algorithm, the boundary of the right lung is achieved. Thus the initialization of lung contour is fulfilled.

#### 2.2.2. Maximum Cost Path-Based Lungs Separation

**Figure 5:**
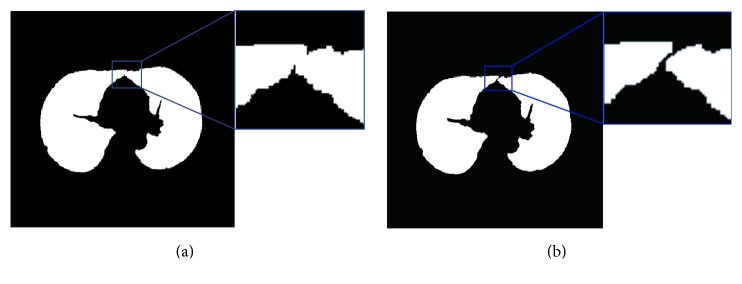
Illustration of left and right lungs separation. (a) Connective case of left and right lungs. (b) Separation of left and right lungs after maximum cost path process.

The separation of left and right lungs is the necessary step for accurate lung segmentation. In [20, 21], 2D edge tracking was used to find the boundaries of the left and right lungs. Hu et al. [22] separated the left and right lungs by identifying the anterior and posterior junctions using dynamic programming. In this paper, we use the dynamic programming algorithm [22] for separation purpose. The dynamic programming algorithm is used on each slice with single connective component. Its target is to locate the position of the left and right lungs and reseparate them (see Figure 5). In this method, the weight map that is proportional to the intensity level is used for searching the maximum cost path, which corresponds to the separation line of left and right lungs.

Once the single connective area is found, 2D erosion process is applied for separation, while dilating process with constraint is used for reconstructing the original borderline. Supposing *A* as the original set of lung pixels, the erosion operation is adopted to calculate a new set for separated lungs *S*. The equation is showed as follows:

(2) $$\begin{array}{c} S=A⊖nB_{4}, \end{array}$$

where ⊖ indicates binary morphology erosion, and *B*_4_ is a binary diamond-shaped structure. By iterative erosion with *B*_4_, *S* is separated into two components, and the iterative number is indicated by *n*.

For the reconstruction of lung border, iterative dilation with constraint is used that is described as follows:

(3) $$\begin{array}{c} C^{i+1}=C^{i}\cup \left\{\;\left\{\;p\;\right\}⊕B_{4}\;\right\}, \end{array}$$

where ⊕ represents morphology dilation, with constraint *p* ∈ *C* ^*i*^∩*A*, while *C* ^*i*^ keeps the same components number with *C* ^*i*+1^, and *C* ^0^ = *S* is used for initialization. Equation (3) is implemented until *p* ∈ *C* ^*i*^∩*A* is not satisfied or the component number is changed. Figure 5 illustrates the reconstruction process.

### 2.3. Pulmonary Parenchyma Refinement

In this section, two successive phases are implemented to refine the rough lung contour. We will describe the details step by step until the final pulmonary parenchyma is achieved.

#### 2.3.1. Arc Reconstruction-Based Border Smoothing

**Figure 6:**
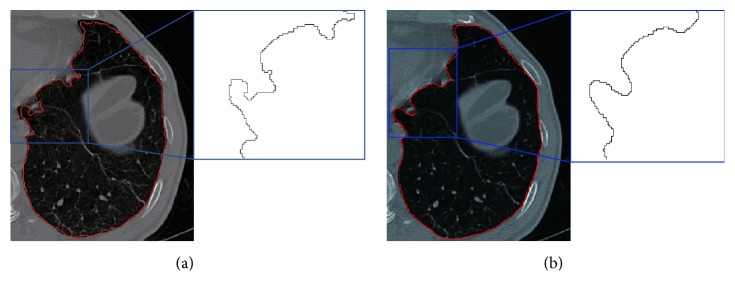
Processing of jagged border. (a) Image with jagged border. (b) Image after smoothing.

Lots of jagged edges are generated after rough segmentation of lungs as shown in Figure 6. In order to make image smooth and reduce the impacts of gradient mutations, curve smoothing method is used. The partial arc coefficient is produced by multiple points, and through appropriate smoothing frequency, the optimum result is obtained. Since any curve on a plane can be defined as *x* = *x*(*s*), *y* = *y*(*s*) (where *s* represents the arc length of the curve), therefore, the edge of the lung parenchyma can be denoted using (4):

(4) x=xs=a0+a1s+⋯+ansn,y=ys=b0+b1s+⋯+bnsn,

(5) Pn−P12≤2,an−a12+bn−b12≤2.

Smoothing is essentially a resampling process, and the convergence condition is (5). When the start points with the last two ones constitute a 8-neighborhood relation, a closed contour is determined. In this paper, cubic spline interpolation [23] is used for constructing the new smoothing border, and the detailed algorithm is described below.

**Step 1 .**

Resampling the initial contour ({*P*_1_, *P*_2_,…, *P*_*N*_}) with step size *L*, and then, the arc length between two adjacent points can be denoted by *L*; in this paper, *L* = 0.3 is used.

**Step 2 .**

For *P*_*i*_ on the border with adjacent points {*P*_*i*−*k*_,…, *P*_*i*−1_, *P*_*i*+1_,…, *P*_*i*+*k*_} (2*k*  (2*k* > *n*)). The arc between the 2*k* + 1 neighbors and *P*_*i*_ are 0,…, (*k* − 1)*L*, *kL*, (*k* + 1)*L*,…, 2*kL*. Assuming (*x*_*i*+*j*_, *y*_*i*+*j*_) as the coordinate of *P*_*i*+*j*_, and *S*_*i*+*j*_ as the arc length between *P*_*i*+*j*_ and *P*_*i*−*k*_, we get the following deduction:

(6) xi+j=a0+a1si+j+⋯+ansi+jn,yi+j=b0+b1si+j+⋯+bnsi+jn.

Then, the least squares method [24] is utilized to obtain the coefficient series *a*_0_, *a*_1_,…, *a*_*n*_, *b*_0_, *b*_1_,…, *b*_*n*_.

**Step 3 .**

Take the arc lengths *s* = *kL* of *P*_*i*_ and *P*_*i*−*k*_ into polynomial, and a new smoothed location $\left(\bar{x_{i}},\bar{y_{i}}\right)$ is generated.

**Step 4 .**

Repeat Steps 2 and 3 for a new border set until convergence.

**Step 5 .**

Set threshold *T*_2_ for perimeter convergence, iterating from Steps 1 to 5 until |*C* − *C*′| < *T*_2_.

The effect on jagged border smoothing is shown in Figure 6, with the testing parameters provided in Table 1. In this paper, parameters *n* = 2, *k* = 4, and *M* = 12 are selected.

#### 2.3.2. Concave Discrimination-Based Border Correction

After border smoothing, appropriate detection and correction are required for solving undersegmentation problem caused by juxta-pleural nodules. The following approach is aiming to this target.

**Figure 7:**
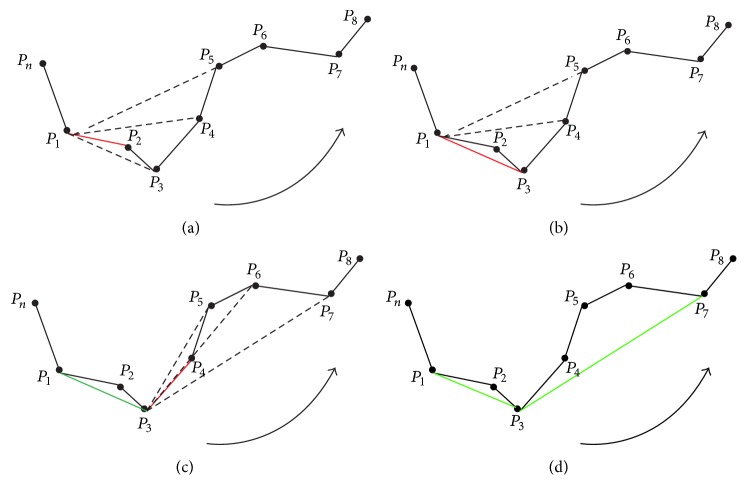
Illustration of border marching algorithm. (a) Start point. (b) Reference direction. (c) New point is found on the right of the reference direction. (d) New reference direction.

**Figure 8:**
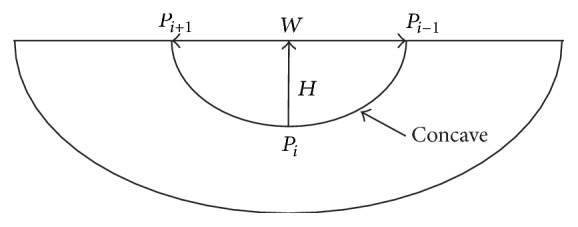
Illustration of the length and width of concave area, where *W* represents width, and *H* represents height.

Concave area is defined as the line between the start point and the rightmost or leftmost point of step size. To determine the orientation, the right hand rule [25] is used, and for detecting all the possible concave areas, the adaptive border marching algorithm (ABM) [13] is utilized. We have developed a model with two parameters (Figure 8) for the determination of boundary refinement. One parameter is *W*, the Euclidean distance between two consecutive points after the marching operation, and the other is *H*, the maximum height perpendicular to this connecting line segment. We defined the threshold which is the length-width ratio of *H* and *W*. For any concave region where threshold > *T*_1_, replace the concave area with a straight line. The ABM algorithm involves five consecutive points, as shown in Figure 7. Choose *P*_1_ as the start point and *P*_1_ *P*_2_ as the reference direction (indicated by red line); then, because point *P*_3_ is found on the right of *P*_1_ *P*_2_, thus, *P*_1_ *P*_2_ is substituted by *P*_1_ *P*_3_ as a new direction. Since all the rest points locate on the left side, thus, a new concave point is detected (indicated by green line). Then, a new round with the new point *P*_3_ is continued until a closed path is achieved.

As concave region detection is completed, we step into the correction phase. On the one hand, concave area correction can reduce the missed diagnosis rate of juxta-pleural nodules; on the other hand, excessive correction will undoubtedly results in more undersegmentation errors. Therefore, length-width ratio-based threshold is proposed for solving this problem. The main procedure of this algorithm is described below.

**Step 1 .**

Calculate the perimeter *C* of lung border set *P*_1_.

**Step 2 .**

For all the concave points on border set *P*_1_, calculate the length-width ratio *η* = *H*/*W* (see Figure 8).

**Step 3 .**

For any concave point that *η* > *T*_1_, substitute the concave area with a straight line, where *T*_1_ indicates the ratio-based threshold.

**Step 4 .**

Recalculate a new lung border set *P*_2_ with perimeter *C*′.

**Step 5 .**

Set the threshold *T*_2_ for perimeter convergence, iterating from Steps 1 to 5 until |*C* − *C*′| < *T*_2_.

**Figure 9:**
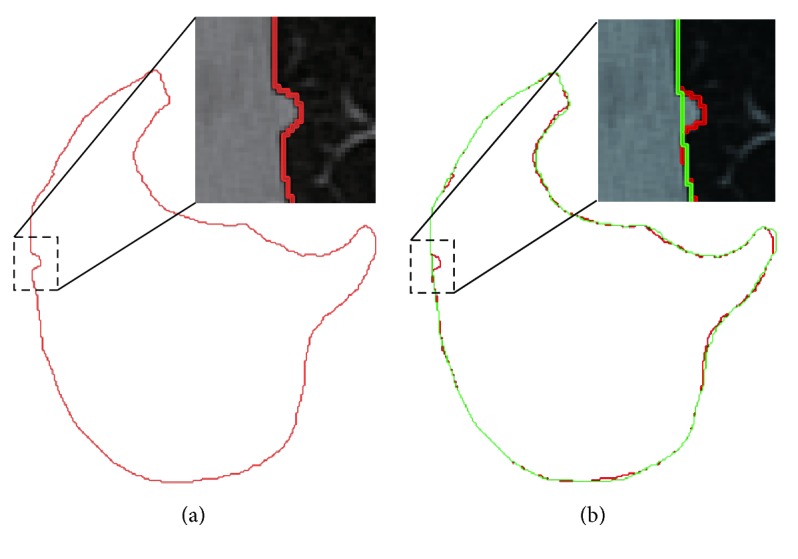
Illustration of border correction. (a) Undersegmentation. (b) After border correction Red line denotes the rough segmentation, while green line represents the effect of correction.

In this paper, the convergence threshold *T*_2_ = 0.01 is used, and Figure 9 depicts the undersegmentation case via concave correction.

## 3. Experimental Results and Discussion

### 3.1. Quality and Accessibility of Datasets

A total of 45 sets of chest CT scans from Weihai Municipal Hospital are used for experiment, in which 20 groups are generated by Somatom Sensation 64 of Siemens Medical Systems, and another 25 groups come from Brilliance 64-bit scanner of Philips Medical Systems. CT size is 512 × 512 × 275 to 512 × 512 × 502, with pixel size 0.625 mm to 0.742 mm, and slice thickness 0.55 mm to 1.0 mm.  Table 2 presents a detailed information about the quality of the images; meanwhile, Table 3 provides a detailed description of juxta-pleura tumor used in our experiment. All 45 groups are manually segmented by a radiologist as golden standard. Experiments are performed in Matlab R2010a, with quad-core CPU i5-4590 and 8 G memory.

The ground truth of the segmentation used in this paper was obtained by the radiologists of the cooperative hospital, utilizing a manual segmentation software named MITK [26], which provides an open source and a graphical user interface developed by the German Center for Cancer Research. The general procedure for ground truth segmentation is as follows. First, a smoothing operation is selected for reducing the noises in the images; second, a three-dimensional region growing method is used to obtain an initial segmentation result; finally, the rough segmentation results were further optimized by the radiologists on the cross-sectional, sagittal, or coronal slice until the final segmentation results were satisfactory.

### 3.2. Evaluation Metrics and Criteria

To evaluate the segmentation performance, seven error metrics are used in this paper, which are often utilized for evaluating on the accuracy and complexity [6, 12], including volume difference (VD), volume overlap error (VOE), relative volume difference (RVD), average surface distance (ASD), root mean square distance (RMSD), maximum symmetric absolute surface distance (MSD), and process time. For automatic segmentation volume *V*_auto_ and manual segmentation volume *V*_manu_, VD is defined as VD = *V*_auto_ − *V*_manu_, RVD = 100 × ((*V*_auto_ − *V*_manu_)/*V*_ref_), VOE = 100 × (1 − (*V*_auto_∩*V*_ref_/*V*_auto_ ∪ *V*_manu_)), and ASD, RMSD, MSD are defined by (7), (8), and (9), respectively:

(7) ASDA,B=1SA+SB∑sA∈SAdsA,SB+∑sB∈SBdsB,SA,

(8) RMSDA,B=1SA+SB×∑sA∈SAd2sA,SB+∑sB∈SBd2sB,SA,

(9) MSDA,B=max⁡maxsA∈SA⁡dsA,SB,maxsB∈SB⁡dsB,SA,

where *A* and *B* correspond to two segmentation results, and *d*(*v*, *S*(*X*)) represents the shortest Euler distance from voxel *v* to the segmentation result *X*.

Similar to the criteria used in [13], oversegmentation and undersegmentation rates are also considered as criteria for comparative study. Oversegmentation rate is defined as the segmentation volume that is regarded as lung tissue in our method, while not in the ground truth, and the undersegmentation rate is vice versa. We use the cumulative distribution to demonstrate the fitting between the lung surfaces obtained by the proposed method and the ground truth, which are calculated by the shortest distance between a point on the lung surfaces obtained by the proposed method and the lung surfaces of the ground truth.

### 3.3. Accuracy Analysis

Table 4 shows the experimental result on 45 chest scans, based on the proposed method and the golden standard. As shown in the table, VD is 11.15 ± 69.63 cm^3^, VOE is 3.5057 ± 1.3719%, ASD is 0.7917 ± 0.2741 mm, RMSD is 1.6957 ± 0.6568 mm, and MSD is 21.3430 ± 8.1743 mm. In clinical practice, VOE of 5% is generally considered as the most acceptable error, and therefore, the proposed method is capable of providing clinical assist.

**Figure 10:**
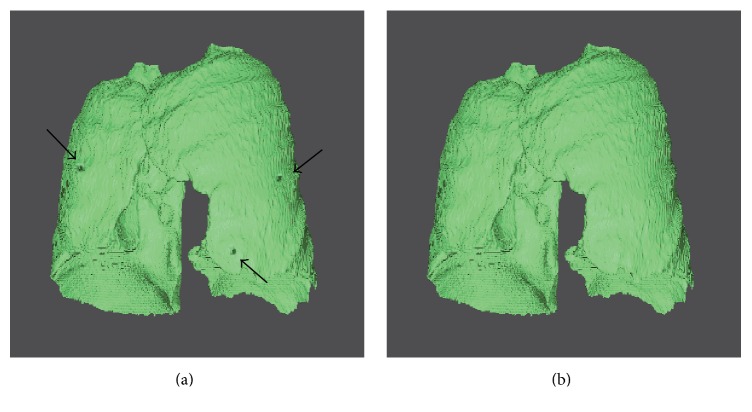
3D view of concave correction. (a) 3D view of juxta-pleural nodules before correction. (b) 3D view of juxta-pleural nodules after correction.

**Figure 11:**
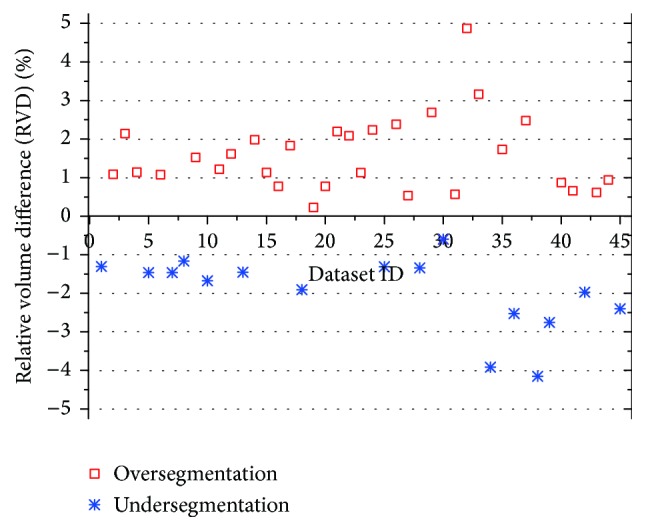
Comparative relative volume difference (RVD) of undersegmentation and oversegmentation.

The automatic segmentation results were compared with manual segmentation result of the radiologist. Whether a juxta-pleural nodule was correctly included or not was determined by a radiologist to see whether there are obvious defects in the segmentation due to juxta-pleural nodules.  Figure 10 shows the three-dimensional view before and after border correction. It can be seen that the juxta-pleural nodules are reincluded after correction operation. However, to a certain extent, oversegmentation error is inevitable due to the overcorrection; thus, most of VD in Figure 11 are positive, which appear above the *x*-axis, denoting oversegmentation. The over- and undersegmentation are 29 and 16 sets, respectively; in other words, the probability of oversegmentation is almost twice of undersegmentation.

**Figure 12:**
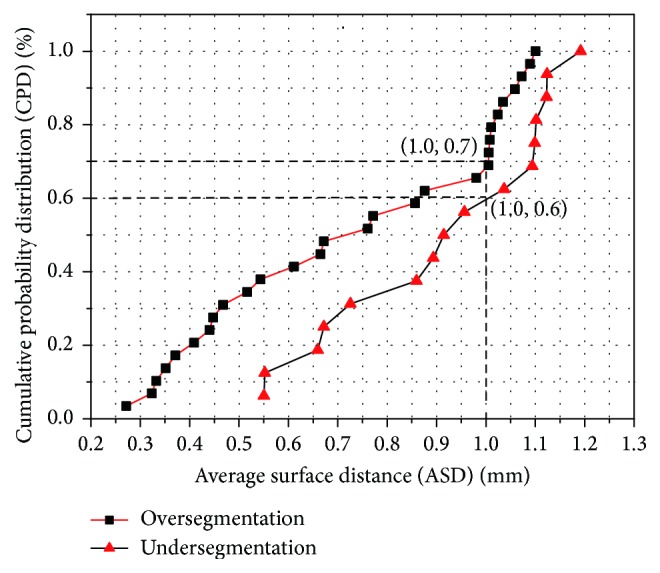
Cumulative probability distribution function of under-segmentation and over-segmentation. (■) 29 groups of over-segmentation. (▲) 16 groups of under-segmentation.

In order to study the average distance of segmentation error, we depict the cumulative probability distribution based on under- and oversegmentation, which are showed in Figure 12. In general, oversegmentation is defined as the lung volume that is regarded as lung tissue in our segmentation method while not in the reference standard, and the undersegmentation is vice versa. In this paper, the metric of RVD (relative volume difference) is used for determining whether a segmentation belongs to oversegmentation or undersegmentation. If RVD gets a positive value, we regard this segmentation result as an oversegmentation on the whole, or undersegmentation vice versa.

We use the cumulative distribution to demonstrate the fitting between the lung surfaces obtained by our method and the manual segmentation standard. The cumulative distance distribution is formed by calculating the metric of ASD (average surface distance) obtained by our automatic method and manual segmentation standard.

In Figure 12, cumulative probability distribution for over- and undersegmentation within 1 mm are 70% and 60%, respectively, while the maximum distance errors are 1.1 mm and 1.2 mm, respectively, thus proving the higher probability of segmentation errors generated by oversegmentation.

### 3.4. Complexity Analysis

**Figure 13:**
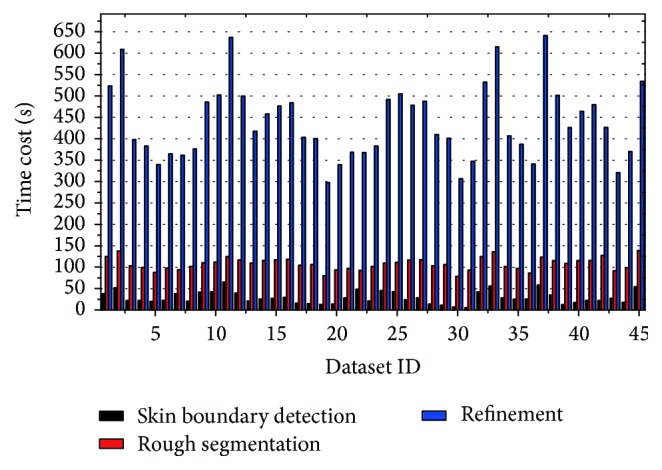
Time-consuming diagram of the main phases of the whole system.

Figure 13 shows the time-consuming diagram of the main processing phases, including skin boundary detection, rough segmentation lung parenchyma, and refinement of lung parenchyma. In the figure, the whole time-consuming is 537.73 ± 162.873 seconds on average, among which skin boundary detection costs 25.2 ± 12.2376 seconds (accounting for 4.66% of the whole time), rough segmentation of lung parenchyma costs 102.45 ± 28.5473 seconds (accounting for 19.03% of the whole time), and refinement costs 408.98 ± 16.788 seconds (accounting for 76.31% of the overall time). And the time complexity for these phases are *𝒪*(*N* ^2^), *𝒪*(*k*_1_ *N* ^2^), and *𝒪*(*k*_2_ *N* ^2^), respectively, where *k*_1_ indicates the iteration numbers of maximum cost path, while *k*_2_ indicates the iterative number of reconstruction and concave determination.

It can be seen from Figure 13 that the proposed scheme spends much more time on smoothing and correction process, in which iterative convergence accounts for a large proportion. On the average, processing time for each image is 2 seconds, while radiologist needs 1 minute for manual segmentation, which proves the efficiency of the proposed scheme.

### 3.5. Comparison with State of the Art Method

To evaluate the performance of our method, the proposed method was compared with the state of the art method proposed by Pu et al. [13]. In Pu's method, an adaptive border marching (ABM) algorithm was proposed to segment the lung and correct the segmentation defects caused by juxta-pleura nodules while minimizing undersegmentation and oversegmentation relative to the true lung border. The primary emphasis and distinguishing characteristic of the proposed method is on robustly correcting missed juxta-pleural nodules.

Table 5 presents the lung segmentation results by using our proposed method on 45 datasets, when compared to the conventional ABM-based method (Pu's method). For the final segmentation results, our method yields mean VOE of 3.51% and ASD of 0.79 mm, while conventional ABM-based method yields mean VOE of 3.86% and ASD of 0.83 mm. Our method outperforms the conventional method by 0.35% and 0.04 mm on average in terms of AOE and ASD, respectively.

**Figure 14:**
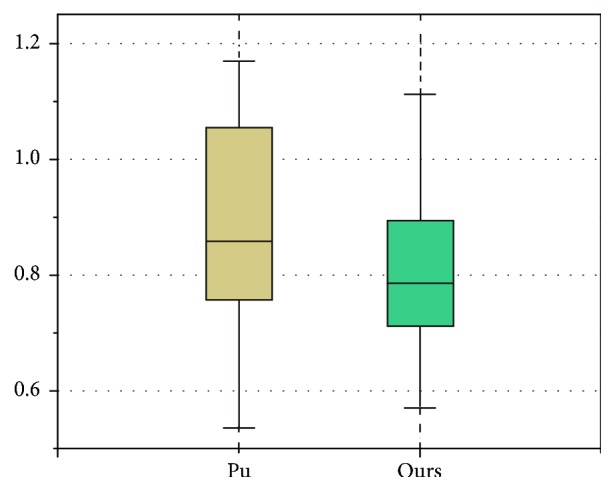
Boxplot on ASD between Pu's and our methods.

In addition, similar results were also obtained for comparison study by boxplot between Pu's and our method in Figure 14. For the ASD, there is no abnormal point in both results; meanwhile, *p* value is less than 0.05 by *t* test, hence proving the significant better accuracy of our method.

Table 6 lists the comparative RVD results on both methods. For the RVD result of oversegmentation, our method and conventional ABM yield mean 1.87% and 1.92%, respectively, while, for the RVD result of undersegmentation, our method and conventional ABM yield mean −1.58% and −1.65%, respectively. Our method outperforms the conventional ABM by 0.05% and 0.07% on average in terms of RVD on oversegmentation and undersegmentation, respectively.

Therefore, for the lung tissue with juxta-pleura nodules, our proposed method achieves more accurate and robust segmentation results than the conventional method. The main reason for that is the lungs separation operation in our method improves the accuracy of lung contour segmentation. It can thus be deployed for accurate and robust lung segmentation with juxta-pleura nodules.

**Figure 15:**
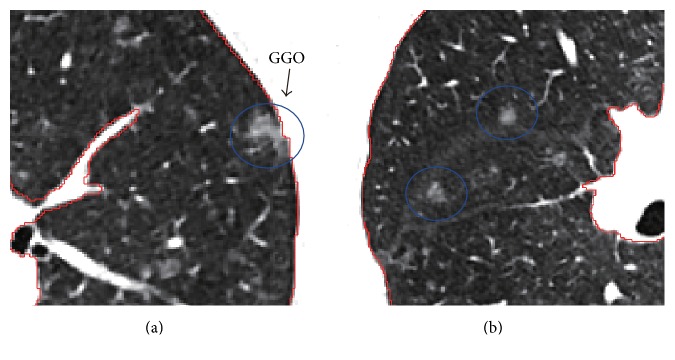
Illustration of lung segmentation result with GGO (ground glass opacity) nodules. (a) Juxta-pleura GGO. (b) Isolated GGO.

For our study, the main target is to solve the problem of the segmentation result by juxta-pleural nodules; thus it is not a generic tool to have this segmentation method when lung includes other pathological lesions or abnormities especially near the pleura. However, in our datasets, GGO (ground glass opacity) nodules are also considered in our scans, some of which are attached to the pleura. Although GGO often shows the irregular shape and low intensity, and its irregular shape is usually not fit to regular concave area detection algorithm, its low contrast with the lung parenchyma helps obtain the correct lung contour. In Figure 15(a) the lung segmentation is performed correctly due to the superiority of border tracing algorithm even when GGO is attached to the pleura, while other conventional region growing-based methods often need further processing because of the inhomogeneity between GGO and the lung parenchyma.

**Figure 16:**
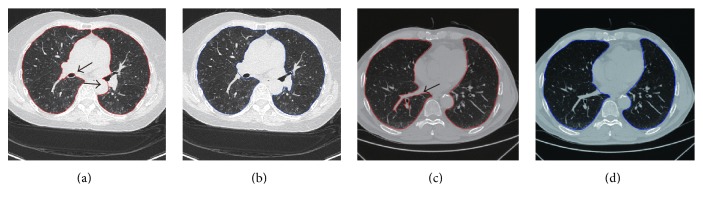
Illustration of segmentation errors of undersegmentation and oversegmentation. (a) and (c) indicate oversegmentation and undersegmentation, respectively. (b) and (d) indicate the manual segmentation as golden standard.

We recognize that the proposed scheme still needs further improvement. As the failure cases Figure 16(a) demonstrated, oversegmentation occurred around the trachea which is close to the parenchyma, and that is the result of overcorrection. In Figure 16(b), when the big vessel is located on the edge of the lung parenchyma, undersegmentation occurred because of the undercorrection. It is difficult to overcome this dilemma through 2D slice since border correction is a trade-off problem. Nevertheless, the segmentation error generated by juxta-pleura nodules could be reduced significantly due to the appropriate length-to-high ratio. Further study on how to reduce the errors caused by trachea and vessel could help alleviate the above-mentioned dilemma.

## 4. Conclusion

In this paper, a fully lung segmentation framework for chest CT with juxta-plural nodules is proposed via five main procedures, including chest segmentation, lung border tracing, left and right lung separation, lung border smoothing, and border correction, which focus on the oversegmentation problem caused by juxta-pleural nodules. Compared with manual segmentation, the volume overlap error of our approach is less than 5%, which meets the clinical requirements. And also, the time-consuming is about 2 seconds per image, which is more efficient than the manual cost of 1 minute per image. However, the proposed scheme tends to result in some undersegmentation, especially around the area which is close to the mediastinum, where the dense tracheas are located. Therefore, the border correction algorithm still needs further improvement, especially for the irregular lung shape caused by abnormal lesions. Nevertheless, comparing with the traditional method, our proposed scheme achieved great advantages in accuracy and time complexity, which indicates a potential tool for lung segmentation with juxta-pleural nodules.

## Figures and Tables

**Table 1:** Parameters in arc reconstruction-based smoothing.

*n* = 1			*n* = 2			*n* = 3			*n* = 4		
*k*	*M*	*t*	*k*	*M*	*t*	*k*	*M*	*t*	*k*	*M*	*t*
1	50	14.2	2	120	23.3	2	120	30.5	3	240	56.6
2	10	5.30	3	30	6.81	3	30	6.57	4	135	43.4
3	8	15.4	4	12	3.25	4	10	2.79	5	50	17.6
4	4	0.78	5	6	1.71	5	6	1.57	6	20	9.10
5	2	0.62	6	4	1.09	6	4	1.15	7	15	5.71

*n* = 5			*n* = 6			*n* = 7			*n* = 8		
*k*	*M*	*t*	*k*	*M*	*t*	*k*	*M*	*t*	*k*	*M*	*t*

3	300	98.06	4	620	252.33	4	800	380.52	5	1000	756.61
4	150	53.76	5	380	176.24	5	600	261.17	6	700	437.78
5	65	27.13	6	190	82.35	6	300	159.47	7	200	127.86
6	30	11.34	7	110	59.07	7	150	76.33	8	90	61.65
7	10	4.11	8	60	28.56	8	70	41.96	9	50	38.12

**Table 2:** Quality and accessibility of the image datasets.

Dataset	Number	Size	Resolution	Slices number	Slice thickness
Local hospital	45	512*∗*512	0.625–0.742 (mm)	275–502	0.55–1 (mm)

**Table 3:** Information of juxta-pleura tumors.

Dataset	Types	Number	Size
Local hospital	Normal nodules; GGO	53	6–17 (mm)

**Table 4:** Experimental result of lung segmentation on 45 testing scans. AVG indicates average result, and SD is short for standard deviation.

ID	VD	VOE	RVD	ASD	RMSD	MSD	*V*_auto_	*V*_manu_
	(cm^3^)	(%)	(%)	(mm)	(mm)	(mm)	(cm^3^)	(cm^3^)
1	−47.31	3.0242	−1.31	1.099	1.7593	18.9573	3671.56	3624.25
2	38.30	2.7479	1.09	0.7605	1.5615	18.122	3485.20	3523.50
3	71.74	3.6033	2.14	1.0078	1.7504	21.177	3275.97	3347.71
4	41.67	1.6723	1.14	0.6651	2.3539	19.6505	3615.24	3656.91
5	−54.48	3.2045	−1.47	1.123	2.0554	28.2471	3769.77	3715.29
6	31.33	3.6496	1.08	0.8762	1.7107	13.6632	2873.39	2904.72
7	−50.56	4.6419	−1.46	0.8933	1.4647	20.7681	3505.04	3454.47
8	−38.36	2.2887	−1.16	0.5502	1.5762	13.2609	3332.93	3294.57
9	75.49	5.2518	1.52	1.0722	1.5287	17.7907	4881.78	4957.27
10	−62.66	3.1748	−1.68	0.9567	1.8444	15.0682	3796.92	3734.26
11	35.49	2.5787	1.22	1.0584	1.6721	13.6448	2881.47	2916.96
12	43.62	1.6744	1.61	1.0107	1.806	19.3329	2658.32	2701.95
13	−49.49	3.1636	−1.45	1.0369	1.5537	10.0705	3459.81	3410.32
14	59.68	5.4911	1.99	0.7718	1.7302	19.3278	2945.88	3005.56
15	45.51	2.748	1.13	1.005	1.6431	19.9296	3967.29	4012.79
16	29.85	3.3606	0.77	0.9802	0.9411	11.3013	3825.41	3855.26
17	64.09	2.4039	1.83	0.4677	0.7013	10.2471	3439.35	3503.43
18	−57.97	4.3397	−1.91	1.1241	1.3929	22.9175	3099.42	3041.45
19	7.98	5.5238	0.23	1.1006	1.9721	15.0682	3531.61	3539.59
20	30.77	3.6862	0.78	1.0243	1.7763	20.1077	3938.13	3968.90
21	82.82	1.7774	2.20	0.3712	0.7476	18.913	3685.22	3768.04
22	78.42	6.1284	2.09	0.5164	0.9187	37.7341	3681.38	3759.81
23	39.89	5.0326	1.13	0.6109	1.8158	30.304	3491.12	3531.01
24	75.16	1.7624	2.24	0.4399	1.1044	17.6525	3283.02	3358.19
25	−50.81	4.5438	−1.31	0.6719	2.3576	32.5025	3918.14	3867.33
26	92.24	5.9488	2.38	1.0348	3.4893	38.3925	3776.06	3868.30
27	15.43	2.3037	0.53	0.3511	0.9273	16.7561	2879.30	2894.73
28	−46.44	2.5115	−1.34	0.5523	1.5216	16.6689	3508.69	3462.25
29	119.99	4.2139	2.69	0.6718	2.0628	29.6165	4342.92	4462.90
30	−29.24	2.8285	−0.60	0.8594	1.8682	24.9089	4887.26	4858.02
31	21.64	1.7654	0.57	0.5437	1.1489	16.5399	3799.97	3821.61
32	148.21	5.2781	4.87	1.0058	2.9211	25.7359	2892.25	3040.47
33	87.09	6.0183	3.16	1.0896	2.5128	21.8625	2668.69	2755.78
34	−168.48	4.5721	−3.91	1.0939	2.3051	21.9811	4472.12	4303.64
35	51.99	1.8947	1.73	0.3234	1.1074	26.6914	2950.36	3002.35
36	−97.87	5.4153	−2.53	1.1019	2.7363	33.2542	3970.86	3872.99
37	122.95	3.7016	2.48	0.8564	1.8731	22.6337	4835.66	4958.61
38	−137.55	5.2533	−4.15	1.1919	3.0683	47.3566	3449.08	3311.53
39	−83.38	2.6188	−2.75	0.6592	1.4036	17.7262	3110.78	3027.40
40	31.08	1.6871	0.87	0.4472	0.9098	13.2845	3543.87	3574.96
41	26.11	2.4515	0.66	0.2712	0.7285	26.0694	3945.08	3971.20
42	−71.33	2.7375	−1.97	0.7256	1.4599	17.6144	3692.75	3621.42
43	22.82	3.3868	0.62	0.4087	0.9147	10.2961	3681.11	3703.93
44	33.29	1.3839	0.94	0.3318	0.7743	12.3896	3505.11	3538.40
45	−77.18	4.3108	−2.40	0.9147	2.8345	34.8965	3290.29	3213.11

AVG	11.15	3.5057	0.3176	0.7917	1.6957	21.3430	3582.57	3593.71
SD	69.63	1.3719	1.9445	0.2741	0.6568	8.1743	529.47	528.37

**Table 5:** Comparative results between our method and the conventional method.

Method	VOE [%]	ASD [mm]	RMSD [mm]	MSD [mm]
Conventional ABM	3.8574 ± 2.10	0.8304 ± 0.33	1.8783 ± 0.51	32.2461 ± 8.12
Our method	3.5057 ± 1.94	0.7917 ± 0.27	1.6957 ± 0.66	21.3430 ± 8.17

**Table 6:** Relative volume difference (RVD) results between our method and the conventional method.

Method	RVD (oversegmentation) [%]	RVD (undersegmentation) [%]
Conventional ABM	1.92 ± 1.02	−1.65 ± 0.93
Our method	1.87 ± 0.95	−1.58 ± 0.96
